# Assessment of self-medication practice and the potential to use a mobile app to ensure safe and effective self-medication among the public in Saudi Arabia

**DOI:** 10.1016/j.jsps.2022.05.010

**Published:** 2022-05-30

**Authors:** Hassan A. Alsaad, Jenan Saleh Almahdi, Nourah Ali Alsalameen, Fadhel Ahmed Alomar, Md. Ashraful Islam

**Affiliations:** a Department of Pharmacology and Toxicology, College of Clinical Pharmacy, Imam Abdulrahman Bin Faisal University, Dammam, Saudi Arabia; b College of Clinical Pharmacy, Imam Abdulrahman Bin Faisal University, Dammam, Saudi Arabia; c Department of Pharmacy Practice, College of Clinical Pharmacy, Imam Abdulrahman Bin Faisal University, Dammam, Saudi Arabia

**Keywords:** Saudi Arabia, Self-medication, Source of information, Arabic medical app, Drug information, Safe medication

## Abstract

**Background:**

Self-medication (SM) plays an essential role in maintaining a good quality of life for individuals. Previous studies suggested that efforts are still needed to ensure the safe practice of SM. The advances in technology and the internet have granted the availability of abundant and easily accessed medication information. However, identifying the reliability of information could be a challenge for the public. This study aimed to investigate the attitude toward SM, determinants of SM, and knowledge about medication in Saudi Arabia. Also, this study aimed to assess the willingness to use a mobile app that would be specifically designed to guide the practice of SM.

**Methods:**

A cross-sectional study was conducted in a form of an online survey among the public in Saudi Arabia. QuestionPro® platform was utilized to collect data from respondents for two months. Statistical analysis was performed using IBM® SPSS® statistics version 26.

**Results:**

A total of 1226 individuals completed the questionnaire. The prevalence of SM practice was 59%. The most frequent reason for not practicing SM was the concern about the drug safety issues (38%). Significant statistical associations were identified between SM and several demographic variables, e.g., age, gender, education, health insurance, and having a chronic illness. Most respondents (82.7%) were aware of the necessity of improving SM practice. Knowledge about different aspects of SM (e.g., proper drug selection and administration) was evaluated based on the consumer’s perspective. Our data showed that overall knowledge about SM was limited for many consumers. The assessment of the participant’s willingness to use a SM app indicated that 47.6% were interested in using such app. This willingness was significantly associated with the consumer’s attitude toward SM and being a chronically ill patient.

**Conclusion:**

SM is a common practice in Saudi Arabia. However, public awareness about SM is limited. So, implementing new strategies to enhance knowledge and ensure the safety of SM is important. A large proportion of participants were interested to use a SM app, which would improve SM practice. Therefore, we recommend developing a SM-oriented app to be used by the public in Saudi Arabia.

## 1 Introduction

Self-medication (SM) is widely accepted as a critical component in the healthcare system. As with other self-care practices such as physical exercise and healthy nutrition, SM aims to maintain the health and wellbeing of individuals or treat a variety of illnesses (Hughes et al., 2001). SM can be defined as the use of medicines to treat self-diagnosed diseases or recognized symptoms or the use of prescribed medicines to treat chronic or recurring diseases by the consumer (WHO, 2000). Here, SM is referred to the selection and use of medicines to treat self-recognized illnesses, without consulting a physician or a pharmacist.

Globally, the prevalence of SM is considered to be high, ranging from 24% to 92% according to a systemic review which included studies that explored the practice of SM among populations in diverse geographical regions (Limaye et al., 2017). During the COVID-19 pandemic, a worldwide upward trend of exploring SM has been reported based on analyzing Google Trends on SM (Onchonga 2020).

Safe and responsible practice of SM among the general public involves using over-the-counter (OTC) drugs, having the appropriate knowledge about the experienced illnesses, and correctly administering drugs. This kind of SM presents many benefits to the individuals and the healthcare systems (Hughes et al., 2001, WHO, 2000). For example, SM can reduce the cost of treatment for patients by buying the needed medicine directly from the community pharmacy and avoiding the cost of consulting a physician. Also, SM can limit the number of visits to hospitals by self-treating minor health conditions at home (Noone and Blanchette 2018). This reduction in the number of patients who visit hospitals is very beneficial in some settings such as the COVID-19 pandemic to avoid spreading the infection and save the government funding and the efforts of the healthcare system to focus on treating serious disease conditions (WHO, 2000).

However, the inappropriate practice of SM through using OTC drugs irresponsibly or using prescription drugs without seeking medical advice from health professionals is considered unsafe SM that could lead to harmful effects to both the individual and the healthcare system (WHO, 2000). SM is associated with risks such as misdiagnosis, ingesting toxic drug doses, long-term use of drug, drug interactions and complications, and polypharmacy (Hughes et al., 2001, Ruiz, 2010). Those risks could leave the disease untreated or worsen the patient’s health condition. Previous studies indicated that unsafe SM is a common phenomenon and actions are needed to minimize this threat to the health of the public (De Bolle et al., 2008, Bennadi, 2013).

Previous research has suggested that the behavior toward SM is influenced by several factors. Some of the factors are related to the demographic characteristics of users such as age, gender, level of education, occupation, and financial income. Other factors are related to the consumers’ knowledge about medicines. In addition, the sources of medicine information such as family, the internet, and social media can influence consumers’ knowledge and the quality of SM practice (Niclós et al., 2018, Amaha et al., 2019, Jember et al., 2019). Based on that, previous research has recommended some strategies to improve the therapeutic outcomes of SM and avoid the harm that is associated with the irresponsible SM. Those strategies have included enhancing the awareness of individuals to gain the appropriate medicine information before practicing SM and implementing new governmental policies to manage the access of medicines for the consumers to ensure the safe practice of SM (Shafie et al., 2018, Tripković et al., 2018, Ansari et al., 2020). Since knowledge about SM is clearly a critical and manageable determinant for the safety of SM, identifying effective approaches that can be applied to enhance the public awareness about medicines would be helpful to improve the quality of SM practice.

While the evolution in technology, especially on the internet and communication devices, has established an easy and quick access to search for knowledge on medicines and diseases as well as SM, work is still needed to ensure the safety and the efficiency of the SM practice (Alpay et al., 2009, Tao et al., 2017). Today, the consumers can find a variety of tools and online resources that expose them to plenty of information that is related to medicines and diseases (Chen et al., 2018). However, some of this information is misleading, unreliable, and cannot be trusted (Daraz et al., 2019). Some of the medicine information is intended to be only used by the health professionals for developing treatment plans but these can be accessed and utilized by the public too. On the other hand, despite of the availability of few medical resources that are intended to be used by the public in Saudi Arabia, those resources provide excess information regarding prescription and nonprescription (i.e., OTC) drugs as well as diseases. Therefore, those resources are not ideal to help individuals in practicing SM since those are not specifically designed to guide the use of OTC drugs for SM (Alduraywish et al., 2020). Furthermore, the appropriate use of technology such as medical mobile apps can lower treatment costs and benefits the economy as reported in a previous study for patients with heart diseases, where the cost-effectiveness analysis was performed to evaluate the impact of apps (Cano Martín et al., 2014). Thus, there is a need to establish a novel tool that can better manage the delivery of medical information to the public to ensure safer, more efficient, and cost-effective SM practice in Saudi Arabia.

The purpose of this study was to assess the attitude toward SM, participants’ perspectives on the quality of SM practice, determinants of SM, and knowledge about medication in Saudi Arabia. Furthermore, this study aimed to evaluate the potential of using a SM app to ensure the safe practice of SM.

## 2 Methods

### 2.1 Study design

A cross-sectional study was conducted in a form of an online survey during the period of January 2021 to February 2021.

### 2.2 Target population and selection criteria

The target population was the general adult public in Saudi Arabia. Inclusion criteria were being at least 18 years of age, able to read Arabic, living in Saudi Arabia, and willing to voluntarily participate in completing the survey. Exclusion criteria were being a physician, a pharmacist, or a student in the field of medicine or pharmacy since these individuals are supposed to be experts in SM and have professional access to medication information.

### 2.3 Sample size

The Raosoft® online calculator was used to estimate the sample size (Raosoft, 2004). Based on the number of the targeted population in Saudi Arabia, a minimum sample size of 1067 was determined to be adequately powered with 3% margin of error, 95% confidence interval, and 50% response distribution.

### 2.4 Recruitment of the study sample and data collection

Using convenience sampling, survey invitations were sent via social media platforms, such as WhatsApp and Twitter, and other internet-based services supplemented by snowballing. The invitation included a brief introduction about the study, eligibility criteria, and a link that directs the interested people to the online survey home page in QuestionPro®, a web‐based survey platform. Responses were collected in QuestionPro® platform. Then, participations were filtered and only the completed surveys were included in the data analysis.

### 2.5 Study instrument

A questionnaire was developed to address the purpose of this study after reviewing the available surveys in the literature. First, the questionnaire was written in English, then it was emailed to several colleagues at the college for feedback. After that, the questionnaire was translated into Arabic language (the primary language in SA) and reviewed by an Arabic-speaking colleague using forward–backward translation method. The questionnaire was pretested (piloted) among ten subjects from the public and later questions were discussed with each subject to ensure face validity. Data gathered from the pilot study were not included in the analysis. The questionnaire was composed of three sections. The first section was concerned with respondents’ demographic information. The second section was intended to assess the practice of SM. The third section was to explore the potential of using a medical Arabic application as a resource to increase awareness of individuals about SM.

### 2.6 Data processing and analysis

The survey data were imported from QuestionPro® to Statistical Package for the Social Sciences (SPSS, IBM Corporation, Version 26) for analysis. Descriptive statistics were performed and expressed as sample counts and percentages. Chi-square test was used to assess the association between different variables. A p-value less than 0.05 was considered statistically significant.

### 2.7 Ethical considerations

The survey started with a brief description of the study. Then, participants were informed that responding to the questionnaire is considered consent for collecting and processing their data confidentially and for research purposes only.

The ethical approval was obtained from the Institutional Review Board at Imam Abdulrahman Bin Faisal University (IRB-2021–05-187).

## 3 Results

### 3.1 Response rate and respondents’ demographics

A total of 1226 respondents had completed the survey (completion rate = 78.5%). About half of the respondents (50.8%) were between 18 and 25 years of age. Most respondents have a Saudi nationality, were not working or studying in the health field-excluding medicine and pharmacy-, did not have any chronic illness, and/or were females (97.06%, 90.22%, 83.4%, and 81.6%, respectively). Around 65% of respondents did not have health insurance, 53.59% lived in the eastern province, and/or 23% hold a bachelor’s degree. More on the descriptive statistics of the demographic characteristics are shown in Table 1.

**Table 1:** Demographic characteristic of respondents (n = 1226).

**Variable**	**Frequency (%)**	
Age	18–25	623 (50.8)
	26–35	370 (30.2)
	36–45	156 (12.7)
	46–55	52 (4.2)
	> 55	25 (2.0)
Gender	Male	225 (18.4)
	Female	1001 (81.6)
Level of education	No formal education	3 (0.2)
	School (elementary, intermediate, or high)	282 (23)
	Diploma	85 (6.9)
	University graduate	736 (60)
	Postgraduate	120 (9.8)
Region	Eastern region	657 (53.6)
	Western region	160 (13.1)
	Central region	312 (25.4)
	Northern region	40 (3.3)
	Southern region	57 (4.6)
Nationality	Saudi	1190 (97.1)
	Non-Saudi	36 (2.9)
Do you have any chronic illness?	Yes	204 (16.6)
	No	1022 (83.4)
Do you have a health insurance?	Yes	427 (34.8)
	No	799 (65.2)
Do you work/study in a health field?^a^	Yes	119 (9.7)
	No	1107 (90.3)

a This study excluded physicians, pharmacists, and students in these two specialties.

### 3.2 Respondents’ practice of self-medication

As shown in Table 2, practicing SM was reported by 723 (59%) of the respondents. The rest of the respondents (n = 503) did not practice SM because they were not sure how to use the medicines safely (38%), were reluctant to use medicines (34.6%), or were not aware that some medicines can be used without consulting an authorized prescriber (26%).

**Table 2:** Self-medication information.

**Component**	**Frequency (%)**	
Have you practiced self-medication? (n = 1226)	Yes	723 (59)
	No	503 (41)
What is the reason for not practicing self-medication? (n = 503)	I do not know that some medicines can be used safely without a medical consultation	131 (26)
	I am not sure how to practice safe SM	191 (38)
	I do not like to use medicines	174 (34.6)
	Others^a^	7 (1.4)

a Selecting this option allowed participants to write their reason. Their responses were, for example, preferring to consult a physician and never needed to take medicine.

Using cross-tabulation, several demographic variables were found to be significantly associated with practicing SM (Table 3). The prevalence of SM was higher among participants who were females, working or studying in the health sector -excluding physicians and pharmacists-, holding health insurance, and/or having a chronic illness. To study the association between SM and the other variables (i.e., age and education), participants were divided into two groups based on the variable being analyzed. With respect to age, SM was more prevalent among individuals who were 18–45 years of age compared to older people. The association between SM and the level of education showed that university graduates and postgraduates had a higher prevalence of SM.

**Table 3:** Cross-tabulation between demographics and SM (n = 1226).

**Variable**		**Practice SM (Yes)**	**P value**
		**Frequency (%)**	
Age	18–45	688 (59.9)	0.013
	> 45	35 (45.5)	
Gender	Male	116 (51.6)	0.012
	Female	607 (60.6)	
Level of education	No school education to diploma	201 (54.3)	0.03
	University graduate and postgraduate	522 (61)	
Do you have any chronic illness?	Yes	135 (66.2)	0.022
	No	588 (57.5)	
Do you have a health insurance?	Yes	235 (55)	0.04
	No	488 (61.1)	
Do you work/study in a health field?^a^	Yes	89 (74.8)	< 0.001
	No	634 (57.3)	

a This study excluded physicians, pharmacists, and students in these two specialties.

**Fig. 1:**
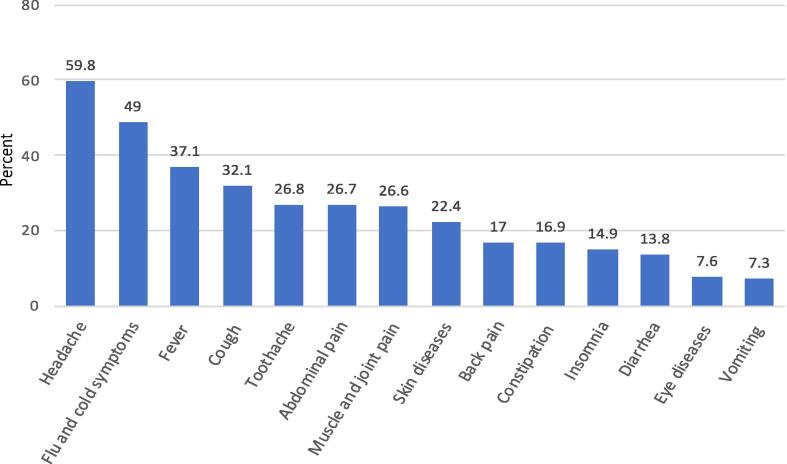
Self-treated health problems and their frequency among respondents (n = 723).

The health conditions that were most commonly self-medicated by participants included headache (n = 432, 59.8%), flu and cold symptoms (n = 354, 49%), fever (n = 268, 37.1%), cough (n = 232, 32.1%), toothache (n = 194, 26.8%), abdominal pain (n = 193, 26.7%), muscle and joint pain (n = 192, 26.6%). More information on the self-treated symptoms or diseases are shown in Fig. 1.

The sources of drug information were investigated to anticipate the safety of SM practice among the participants. The most frequently used sources were experience from previous treatment (65.01%), family and friends (46.61%), and searching on the internet (41.77%). The other sources of drug information such as medical apps were less frequently used in SM (Table 4).

**Table 4:** Source of drug information and attitude toward drug information leaflet.

**Component**	**Frequency (%)**
**Source of information (n = 723)**	
What source(s) of information have you used in practicing self-medication?	
Social media	106 (14.7)
Exploring the web / internet	302 (41.8)
Advertisements	8 (1.1)
Family / friends	337 (46.6)
Experience from previous treatment	472 (65.3)
Medical apps	124 (17.2)
**Attitude toward drug information leaflet**	
How often do you read the drug pamphlet that comes with the drug package? (n = 1226)	
Always	521 (42.5)
Usually	213 (17.4)
Often	168 (13.7)
Sometimes	201(16.4)
Rarely	80 (6.53)
Never	43 (3.51)
How much of the pamphlet information do you understand? (n = 1183)	
Fully understood	550 (46.5)
Partly understood	612 (51.7)
Did not understand at all	21 (1.8)
What challenge(s) have you faced when reading the pamphlet? (n = 1183)	
The font is too small	744 (62.9)
The information is too much	643 (54.4)
The information is difficult to understand	165 (13.9)
None	135 (11.4)
**Improving SM: participants’ perspective**	
What do you think regarding the following statement: Knowledge and practice of SM in Saudi Arabia should be improved. (n = 1226)	
Strongly agree	636 (51.9)
Agree	377 (30.8)
Undecided	185 (15.1)
Disagree	17 (1.4)
Strongly disagree	11 (0.9)

The participants’ reading of the drug information leaflet was explored to evaluate its effectiveness in promoting safe SM. A total of 521 respondents (42.5%) reported that they always read the drug information leaflet, and 43 respondents (3.51%) never read it. Only 550 (46.49%) of the respondents who read the leaflet understood all the inserted information. Also, respondents who read the leaflet were asked about the challenges they face when reading the leaflet. The most frequent challenges were the small font and the extensive information which were reported by 744 (62.89%) and 643 (54.35%), respectively (Table 4).

The participants’ satisfaction with SM was investigated to understand their perspective regarding the practice of SM. Only 152 (21%) of the respondents who practiced SM were fully satisfied with SM. Moreover, all the respondents (n = 1226) were asked whether SM needs to be improved in Saudi Arabia. Notably, most respondents (82.6%) strongly agreed/agreed with the need for improvement of SM (Table 4).

### 3.3 Respondents’ knowledge about self-medication

The questionnaire aimed to assess knowledge of self-medicating respondents about medicines and SM practice. Out of those who practiced SM (n = 723), the findings indicated that only 147 (20.3%) of respondents were confident that the most appropriate medicine was used in SM, 273 (37.8%) were confident that the used medicine did not need a medical consultation, and 27 (37.5%) had the adequate knowledge about the appropriate administration of the medicine. Surprisingly, it was found that 464 (64.2%) of participants were not aware that the same drug can be sold under different brand names. More information regarding the evaluation of participants’ knowledge is shown in Table 5.

**Table 5:** Medication knowledge (n = 723).

**Knowledge Component**	**Frequency (%)**		
	**Confident**	**Somewhat confident**	**Not at all confident**
1. How confident were you in self-diagnosing health problem accurately?	160 (22.1)	477 (66)	86 (11.9)
2. How confident were you that the used drug is the best choice to treat the health problem?	147 (20.3)	466 (64.5)	110 (15.2)
3. How confident were you that the used drug does not need a medical consultation before use?	273 (37.8)	328 (45.4)	122 (16.9)
4. How confident were you that whether the used drug should be taken with food or on an empty stomach?	211 (29.2)	305 (42.2)	207 (28.6)
5. How confident were you about the appropriate use of the drug (*e.g.,* dose, frequency)?	271 (37.5)	385 (53.3)	67 (9.3)
6. How confident were you about the side effects of the used drug in self-medication?	216 (29.9)	314 (43.4)	193 (26.7)
	**Yes**	**No**	
7. Are you aware that the same drug can be sold under different brand names?	259 (35.8)	464 (64.2)	
8. Are you aware that some drugs should not be taken in some chronic diseases?	262 (36.2)	461 (63.8)	

### 3.4 Willingness to use an Arabic SM-related mobile app

To determine the potential benefit of apps in improving the practice of SM, the willingness of participants was assessed by a set of questions in the survey. Out of 1226 participants, 584 (47.6%) were willing to use a SM app, 519 (42.3%) perhaps would use it, and only 123 (10%) were reluctant to use such app. Around half (51.2%) of the participants in the first two groups (i.e., willing and possibly willing) would have access to the app free of charge (Table 6).

**Table 6:** Willingness to use an Arabic medical app for practicing SM.

**Component**	**Frequency (%)**			
Would you use a medical app that is designed to help improve SM practice? (n = 1226)	Yes	Possibly	No	
	584 (47.6)	519 (42.3)	123 (10)	
How much would you pay to access an app for SM per year? (n = 1103)	100 SR	50 SR	10 SR	0 SR
	63 (5.7)	266 (24)	209 (18.9)	565 (51.2)

**Fig. 2:**
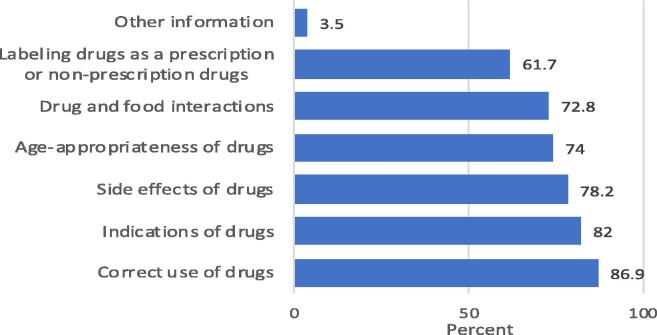
Important drug information according to respondents (n = 1103).

Regarding the drug information, most participants (86.9%, 82%, and 78.2%) were interested to have information about the correct administration, indications, and side effects of medicines, respectively. More information in this regard is shown in Fig. 2.

Cross-tabulation was used to determine associations between the willingness to use a SM app and participants’ demographics and association between the willingness and participants’ attitude toward SM. It was found that practicing SM, having a chronic disease, and agreeing with the need to improve SM were significantly associated with the willingness to use the app (Table 7).

**Table 7:** Factors influencing the willingness to use a SM-oriented app (n = 1226).

**Variable**		**Willing to use the app (Yes)**	**P value**
		**Frequency (%)**	
Age	18–25	308 (49.4)	0.446
	26–35	174 (47)	
	36–45	67 (42.9)	
	46–55	26 (50)	
	> 55	9 (36)	
Gender	Male	99 (44)	0.128
	Female	485 (48.5)	
Level of education	No formal education	2 (66.7)	0.6
	Schools	124 (44)	
	Diploma	44 (51.8)	
	University Graduate	355 (48.2)	
	Postgraduate	59 (49.2)	
Do you have any chronic illness?	Yes	111 (54.4)	0.02
	No	473 (46.3)	
Do you have a health insurance	Yes	200 (46.8)	0.364
	No	384 (48.1)	
Do you work/study in a health field?^a^	Yes	59 (49.6)	0.363
	No	525 (47.4)	
Have you practiced self-medication?	Yes	383 (53)	< 0.001
	No	201 (40)	
What do you think regarding the following statement: Self-medication practice in Saudi Arabia should be improved.	Strongly agree	368 (57.9)	< 0.001
	Agree	158 (41.9)	
	Undecided	51 (27.6)	
	Disagree	5 (29.4)	
	Strongly disagree	2 (18.2)	

a This study excluded physicians, pharmacists, and students in these two specialties.

## 4 Discussion

Previous studies provided insights into the practice of SM in Saudi Arabia. However, the target population in most studies were university students (Al Essa et al., 2019, Alshahrani et al., 2019, Mannasaheb et al., 2021) or the public in one city (Ansari et al., 2020, Islam et al., 2020, AlGhofaili, 2021). This study targeted the adult public across Saudi Arabia. Our finding indicated that the prevalence of SM among the public in Saudi Arabia was 59%. It is worth mentioning that the collection of data was done during the COVID-19 pandemic, so the determined prevalence here may be higher than the prevalence of SM before the pandemic. This is likely because headache, flu and cold symptoms, and fever are common symptoms of COVID-19 infection (Chilamakuri and Agarwal 2021) and the main self-treated symptoms reported in this study. Nevertheless, previous studies in Saudi Arabia identified lower prevalence of SM as reported in Dammam (52.8%) and higher prevalence of SM as reported in Qassim (75.2%) and Majmaah (93.1%) (Alzahrani et al., 2015, Islam et al., 2020, AlGhofaili, 2021). This variation in the reported prevalence may be due to differences in the data collection plan, e.g., the method of sample recruitment and the targeted population. The main reason for not practicing SM was found to be the uncertainty of respondents to use the medicines safely (38%). Our finding is comparable to the reported reasons from a previous study in Ethiopia where 42.6% of participants were worried about using the wrong drug or incorrectly administering the drug (Shafie et al., 2018).

The results of our study showed statistically significant associations between practicing SM and age, gender, education, and occupation. These results are similar to previous findings in Hail (Ansari et al., 2020) and in contrast to the reported findings in Qassim, where purchasing OTC drugs was not significantly associated with age, gender, and educational level (AlGhofaili 2021). In this study, SM practice was found to be significantly associated with having a chronic illness, which is in line with the findings from previous studies in Riyadh, Serbia, and Addis Ababa (Alghanim, 2011, Shafie et al., 2018, Tripković et al., 2018). Our study showed a significant association between SM and health insurance status (*p* = 0.04). Similarly, a previous study suggested that there is an association between SM and the lack of health insurance (*p* = 0.05) (Al-Ghamdi et al., 2020).

The findings here indicated that the most self-treated health conditions were headache, flu and cold symptoms, and fever. Similarly, these symptoms were reported as common self-treated conditions in previous local and worldwide studies (Limaye et al., 2017, Ansari et al., 2020).

The most commonly used source of drug information in this study was the experience from previous treatment. A similar finding was reported in a study from Ethiopia if the consultation of health professionals was excluded from their list. (Shafie et al., 2018). To the best of our knowledge, this is the first study that explored the use of medical apps in SM. The use of medical apps as a source of information was only indicated by 17.2% of the participants, which may be due to the lack of Arabic SM apps. Interestingly, the willingness to use an Arabic SM guide highlights the potential benefit of implementing the proposed method to improve the practice of SM.

The assessment of the participant’s attitude toward the drug information leaflet showed that 42.5% of participants always read the leaflet. This finding is consistent with previous studies from Riyadh (Aljadhey et al., 2015) and Dammam (Islam et al., 2020), where 43.7% and 43.1% of participants indicated that they always read the leaflet, respectively. According to the participants, the most frequent challenges in reading the drug information leaflet were the small font and the extensive information. On the other hand, only 13.9% of participants reported difficulty in understanding the leaflet which is in line with the finding of a previous study in Dammam (7.2%) (Islam et al., 2020). It is important to consider these participants’ perspectives when developing a SM app to enhance its use and positive impact on SM practice.

This study reported that most participants (82.7%) recognized the importance of improving the public awareness and practice of SM in Saudi Arabia. Our finding is in line with previous studies from Medina and Hail cities where the minority of participants (21% and 33.5%, respectively) agreed that SM is a safe practice in Saudi Arabia (Ansari et al., 2020, Allam and Amer, 2020).

The investigation of participants’ knowledge about SM indicated that a limited number of participants were confident to have sufficient information to ensure the proper use of drugs. Consistent with this finding, previous studies from Dammam and Riyadh suggested that the level of knowledge about SM varied among consumers and did not ensure safe medication use for many of them (Aljadhey et al., 2015, Islam et al., 2020).

The evaluation of participants’ willingness to use a SM-oriented app showed that at least 47.6% of participants would use such app to improve their practice of SM. Almost half of the participants (48.8%) were willing to pay for a SM app. This finding is higher than the reported finding in a previous study where only 17% of the participants agreed in spending money on online healthcare services (Al-Anezi 2021). This variation in findings may be because the previous study investigated the participants’ interest in medical consultations such as calling a physician.

The willingness to use a SM app was found to be significantly associated with practicing SM and recognizing the need to improve SM. These results could not be compared to the literature because, to our knowledge, previous studies did not explore the determinants of adopting online resources in SM practice. Our findings also showed that participants’ readiness to use a SM app was associated significantly with having a chronic disease. This is supported by other studies which investigated the use of medical apps in managing chronic diseases such as chronic obstructive pulmonary disease and diabetes (Zhang et al., 2019, Kooij et al., 2021).

## 5 Conclusion

SM was found to be prevalent among the public in Saudi Arabia. Many of the self-medicating participants had limited knowledge about the used medicines. Consistent with that, most participants agreed that it is important to enhance knowledge about medications and improve the practice of SM. Interestingly, a large proportion of participants would use a SM app to guide them in practicing SM. Thus, developing the proposed app and promoting its use among the public will improve the practice of SM in Saudi Arabia.

## Funding

This research did not receive any specific grant from funding agencies in the public, commercial, or not-for-profit sectors.

## Declaration of Competing Interest

The authors declare that they have no known competing financial interests or personal relationships that could have appeared to influence the work reported in this paper.
